# Cost-Effectiveness of a Family Planning Voucher Program in Rural Pakistan

**DOI:** 10.3389/fpubh.2017.00227

**Published:** 2017-09-22

**Authors:** Edward Ivor Broughton, Waqas Hameed, Xaher Gul, Shabnum Sarfraz, Imam Yar Baig, Monica Villanueva

**Affiliations:** ^1^International Health, Bloomberg School of Public Health, Johns Hopkins University, Bethesda, MD, United States; ^2^University Research Co. (United States), Bethesda, MD, United States; ^3^Marie Stopes Society, Karachi, Pakistan; ^4^PMU Punjab Health Agency, Lahore, Pakistan; ^5^Sindh Government Children Hospital, Karachi, Pakistan; ^6^United States Agency for International Development, Washington, DC, United States

**Keywords:** contraception, family planning services, Pakistan, cost-effectiveness analysis, rural health services

## Abstract

**Introduction:**

This study reports on the effectiveness and efficiency from the program funder’s perspective of the Suraj Social Franchise (SSF) voucher program in which private health-care providers in remote rural areas were identified, trained, upgraded, and certified to deliver family planning services to underserved women of reproductive age in 29 districts of Sindh and 3 districts of Punjab province, Pakistan between October 2013 and June 2016.

**Method:**

A decision tree compared the cost of implementing SSF to the program funder and its effects of providing additional couple years of protection (CYPs) to targeted women, compared to business-as-usual. Costs included vouchers given to women to receive a free contraceptive method of their choice from the SSF provider. The vouchers were then reimbursed to the SSF provider by the program.

**Results:**

A total of 168,206 married women of reproductive age (MWRA) received SSF vouchers between October 2013 and June 2016, costing $3,278,000 ($19.50/recipient). The average effectiveness of the program per voucher recipient was an additional 1.66 CYPs, giving an incremental cost-effectiveness of the program of $4.28 per CYP compared to not having the program (95% CI: $3.62–5.31).

**Conclusion:**

The result compares favorably to other interventions with similar objectives and appears affordable for the Pakistan national health-care system. It is therefore recommended to help address the unmet need for contraception among MWRA in these areas of Pakistan and is worthy of trial implementation in the country more widely.

## Introduction

In 2012, the London Summit on Family Planning proposed the ambitious “FP2020” commitment to increase coverage of modern contraceptive methods (MCMs) for 120 million more women and girls by 2020 (1). To work toward this commitment, Marie Stopes International (MSI) has implemented interventions to increase access to modern contraception through methods including social franchising in low-income settings where the unmet need for contraception is highest (2).

Pakistan initiated family planning (FP) programs first in 1953 in the private sector and then in the 1960s in the public sector. However, the contraceptive prevalence rate increased by only 0.25% annually until 1990 when it rose more sharply but still remained behind other countries in the region (3). Pakistan continues to have a high unmet need for FP with a contraceptive prevalence reported as low as 35% among urban woman and 23% in the rural population (4). Improving availability, access, and quality of FP and reproductive health (FP/RH) services for women appears to contribute to addressing the problem in this setting (5, 6).

The Unites States Agency for International Development (USAID) has supported interventions to increase coverage and improve the quality of FP/RH services in Pakistan. One intervention is the Suraj Social Franchise (SSF) implemented by MSI, in which private providers working in remote rural areas are identified, trained, certified and inducted into the franchise. MSI conducted a similar social franchise implementation in Mali from 2012 to 2015. It was successful in this setting in improving FP access, choice and use of contraception among the rural poor (7).

In the Pakistan SSF program, the quality of FP/RH services delivered is improved by a continuous supportive supervision mechanism. From 2013, with support from USAID, Marie Stopes Society (MSS) Pakistan (MSI’s country affiliate) used vouchers that allowed married women of reproductive age (MWRA) to access FP/RH services that are free at the point of service (5). The purpose of this study was to report on the effectiveness and efficiency from the program funder’s perspective of this intervention as implemented in 29 districts of Sindh and 3 districts of Punjab province between October 2013 and June 2016. Such information is useful to donor funders and the Ministry of Health in Pakistan to aid decision-making for investments in FP/RH.

## Intervention

The Unites States Agency for International Development has supported its implementing partner MSS to expand the social franchising network to promote RH/FP services to poor and underserved communities in 32 districts of Pakistan. The franchise is a partnership through which MSS gives training and supervision to private local health service providers, enabling them to provide high quality services to eligible low-income and otherwise disadvantaged women. The women are given vouchers, through door to door visits and support group meetings by the Field Health Educators, that they can redeem for RH/FP services without additional out-of-pocket costs. This facilitates demand for the services while still allowing women to choose their service provider from among the many who are participating in the program. Primary targets of the intervention are MWRA (aged 15–49 years) who live in rural and peri-urban areas with high unmet need of FP and identified as disadvantaged on the Progress out of Poverty Index tool (8).^1^

All SSF facilities underwent third-party post-training evaluation to ensure they were adequately staffed and equipped to provide RH/FP services to national standards. One type of facility, SSF-As, provides short- and long-term contraception methods while SSF-A+ facilities have doctors on staff and available to provide contraceptive implants and tubal ligation in addition to the short- and long-term methods SSF-As can provide. Between 2013 and 2016, MSS inducted 255 SSF-As and 45 SSF-A+ to make a total of 301 SSFs in the 29 districts in Sindh and 3 districts in Punjab. They also provided technical assistance to those providers who required it to become eligible for inclusion as an SSF service delivery center. All contraceptive supplies were provided to women with vouchers and MSS provided ongoing mentoring and supervision at the facilities to maintain high standards of service delivery.

## Method

**Figure 1:**
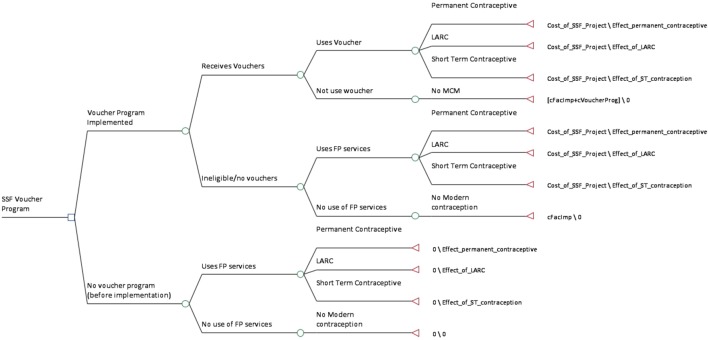
Decision tree for cost-effectiveness analysis of SSF program. Abbreviations: LARC, long-acting reversible contraceptive; FP, family planning; SSF, Suraj Social Franchise; ST, short term; MCM, modern contraceptive method.

A decision tree was constructed to model the program in operation in the 32 districts (Figure 1). Such models are commonly used for economic analysis of FP/RH programs in LMICs (9). We used a single iteration model considering the program’s operation since October 2013 to June 2016 using mostly empirical data from its implementation (Tables 1 and 2). Costs are reported in 2015 US dollars. The model was populated with data collected on costs and the effects of the program on the outcome of interest, namely the additional CYPs attributable to the intervention. To account for the uncertainty in the inputs into the model, we used binomial distributions and ran Monte Carlo Simulations to give a point estimate result with resulting confidence interval.

**Table 1:** Model input variables.

Category	Description	Input value
Costs (US$)	Travel to facilities	1,374,616
	Upgrading equipment at facilities	50,661
	Training health workers	703,635
	Improving family planning facilities	(Sum of above)
	Voucher program (management and distribution of vouchers)	770,985
	Permanent contraceptive method voucher per procedure	38.1
	Long-acting reversible contraceptive (LARC) method voucher	5.29
	Short-term (ST) contraceptive method voucher	0.9
Effects	Couple years of protection for permanent contraceptive	10
	Couple years of protection of LARC methods	4.56
	Couple years of protection of ST contraceptive methods	0.19
Number	All married women of reproductive age voucher recipients	168,206

**Table 2:** Model input distributions.

Binomial distribution probability model inputs	Point estimate
Woman is eligible for a voucher in target area	0.722
Voucher-eligible woman uses vouchers	0.931
Woman ineligible for vouchers uses family planning (FP) services	0.197
Woman seeking FP services chooses long-acting reversible contraceptive (LARC) method	0.188
Voucher-recipient woman chooses a permanent contraceptive method	0.020
Woman is using FP services in the pre-program period	0.231
Woman chooses permanent contraceptive method if not voucher eligible (10)	0.0004
Woman chooses LARC method if not voucher eligible	0.001
Woman seeking FP services in pre-intervention period chooses permanent contraceptive method	0.0001
Voucher-recipient woman chooses an LARC method	0.476

This study relied solely on data already collected during routine monitoring and evaluation of the program and was anonymized for use in this study. Because no additional data were required from human subjects, it was considered exempt from full Institutional Review Board application in the US by University Research Co. Inc. and in Pakistan by the MSS.

### Costs

We used activity-based costing to estimate the resources consumed in implementing this program. Inputs for program costs were compiled from the administrative records of the implementation available from MSS Pakistan. They were divided into (1) voucher management cost, (2) facility support costs, and (3) FP consultation costs.

Social franchise program costs included all staff time, transportation, and administrative costs involved in getting the vouchers to the recipients.

Facility support costs included the time taken by the project staff to conduct the training in participating facilities to ensure they meet the standards of quality service to be part of the program. It also included the cost of the upgrades in equipment and supplies to the facilities required to meet the standards, clinical monitoring and refresher training courses and supervisory visits. No building or infrastructure changes were made to the facilities.

Consultation and service costs for those receiving FP services were mostly captured in the amount redeemed by the private health-care provider using the voucher for payment for the services they rendered. It did not include the cost of contraceptives supplied because these were not borne by the project but many of the supplies were received through the USAID Deliver Project.

Baseline contraceptive prevalence was obtained from a study of coverage in rural Pakistan (10).

### Effectiveness

Effectiveness measures for the program were primarily the uptake of contraceptive methods by MWRA who were eligible for the vouchers. This was measured by MSS as part of their routine monitoring and evaluation system. Secondary measured were couple years of protection (CYP). Both of these were derived from the records of contraceptive uptake determined from redemption of the vouchers. Records of these were recorded and collected in each of the participating providers. For example, if one married woman of reproductive age redeemed a voucher at a participating SSF-A for a tubal ligation, the service provided would be assigned 10 CYPs to represent the what is attributed to that form of contraception in CYPs (11, 12). We assumed that all of the vouchers reimbursed were for FP/RH services that would not have been taken up in the target population without the SSF program.

## Results

A total of 168,206 women of reproductive age received the FP vouchers in this program over the period between October 2013 and June 2016 for a total cost of $3,278,000. The average cost of the program per woman who received the services was $19.50. This includes the cost of upgrading the facilities providing the services divided by the total number of voucher recipients using services at those clinics. The average effectiveness of the program per voucher recipient was an additional 1.66 CYPs. This gives an incremental cost-effectiveness of the program of $4.28 per CYP compared to not having the program (95% CI: $3.62–5.31).

One-way sensitivity analysis was conducted on all of the major input variables to determine their relative effect on the overall results. We increased the values one at a time by 10% to determine the change and magnitude of the incremental cost-effectiveness ratio (Table 3). For example, if the probability that a voucher-eligible woman uses a voucher for FP services or products increases by 10%, holding other variables constant, the program would be approximately 5.45% more cost-effective.

**Table 3:** One-way sensitivity: effect on result with changes in input distributions.

Input probability distribution variables increased by 10%	Change (%)	Direction of change
Voucher-eligible woman uses vouchers	5.45	More cost-effective
Voucher-recipient woman chooses a long-acting reversible contraceptive (LARC) method	5.45	More cost-effective
Woman seeking family planning (FP) services chooses LARC method	0.27	More cost-effective
Voucher-recipient woman chooses a permanent contraceptive method	0.27	More cost-effective
Woman is eligible for vouchers	0	No substantive change
Woman ineligible for vouchers uses FP services	0	No substantive change
Woman chooses permanent contraceptive method if not voucher eligible	0	No substantive change
Woman chooses LARC method if not voucher eligible	0	No substantive change
Woman seeking FP services in pre-intervention period chooses permanent contraceptive method	0	No substantive change
Woman seeks FP services in the pre-program period	0.27	Less cost-effective

**Figure 2:**
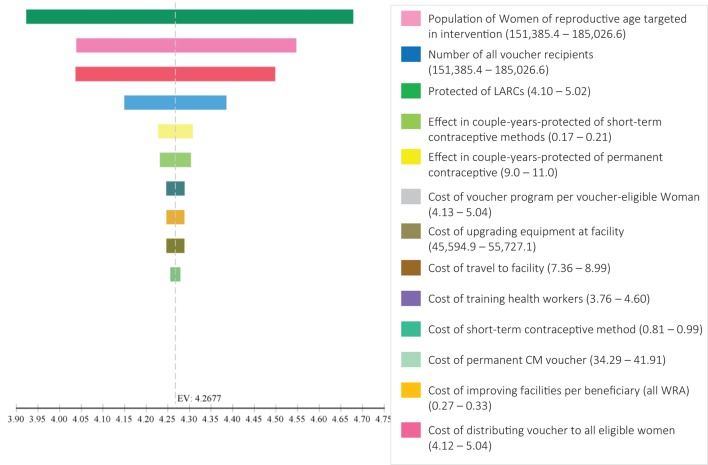
Incremental cost-effectiveness ration tornado diagram for sensitivity analysis of input variables.

One-way sensitivity was also done on other variables in the model that were varied within their feasible range to determine their effect on the overall result (Figure 2). For example, if the CYPs for LARCs varied between 4.1 and 5.2 [the point estimate used was 4.56 based on published conversion tables (11)], the cost-effectiveness of the intervention would range between $2.40 and $4.20 per additional CYP.

## Discussion

Interventions to increase access for women to MCMs in LMICs is important for reaching global health goals but there are few studies on the cost-effectiveness of such interventions (9). This study is an important contribution on the topic. More than 168,000 women in 32 districts of Sindh and Punjab Province received FP services through the voucher program between October 2013 and June 2016 at a total cost of $3,278,000, including the cost of paying for the vouchers redeemed for payment by the participating facilities. This is a total cost for two years of $19.50 per recipient MWRA and an incremental cost-effectiveness of $4.27 per additional CYP achieved by the program compared to business-as-usual. The cost per recipient was less than that reported in 2011 per recipient in Pakistan in each of the four categories (per person served) the authors examined (franchise $31, government $39, private $30 and NGO $24) (13). It was also less than the $55 per woman serviced report in 2013 and the incremental cost per additional CYP is less than the total cost per CYP of $17 in a study also conducted in Pakistan on substantively different programs to this SSF but with the same (14). Using the estimated GDP per capita of $4,600 and estimated health spending of 2.6% giving a total of $119.60/year, this program cost approximately 8% of total health spending per person (15). It suggests the program is affordable overall for the government to provide so that it is free at the point of service for eligible MWRA. Internationally, it compares well against an economic analysis of vasectomies in India from 2007 which showed a cost-effectiveness of $1.31–1.52/CYP (16). It also comparable to the total cost per CYP of contraception in 13 USAID priority countries, which ranged from $2 to $13, depending on the contraceptive method used (17).

The perspective taken for this evaluation was the funder’s—USAID—and it was conducted to inform decision-making by current and potential future external funders. If the analysis was considered from the societal perspective, other costs and consequences of the program would need to be considered. These include additional costs for the contraceptive supplies and the time cost for the MWRA seeking services. On the potential cost-saving side, there would be the decrease in expenditure of the health system due to fewer pregnancies occurring, especially unwanted or high-risk pregnancies that would be avoided when the problem of unmet contraceptive need is satisfied with implementation of the program. Considering the results from this study, it is expected that using this alternative perspective would make the program even more cost-effective than it appeared from the external funder’s point of view. Therefore, we recommend implementation of this program to the provincial or national government if external funding cannot be secured because of its affordability and efficiency and because its benefits accrue to society.

From the tornado diagram and the one-way sensitivity analysis in Table 3, it appears that changes in the costs would not have a significant impact on the overall result. We can therefore surmise that even if implementation was not as efficient as shown here, implementation would still be recommended if the level of effectiveness was achieved.

It is possible that the volume of MWRAs could be increased improving outreach in the region targeted by the intervention or by increasing the geographical coverage without substantive changes in at least some of the administrative and capital costs to gain from economies of scale. It is also possible that some changes in the program, possibly with more behavior change communication, while respecting the client method of choice, to encourage permanent contraception methods or LARCs that last longer would increase the CYPs per consultation which would also improve the cost-effectiveness of the program (18).

It is estimated that there are 50 abortions per 1,000 women in Pakistan or a total of 2.2 million annually (19). It is also reported that one of the main reasons for Pakistani women seeking abortion is the unmet need for contraception services in the country (20). Induced abortions in this setting are often associated with morbidity and mortality at higher rates that in settings where access to appropriate health care is better (21). It was beyond the scope of this study to consider the effects on the rate of abortion in target population for this SSF program. However, it is reported that better access to modern contraception reduces the prevalence of unsafe abortions at this is a positive effect of the intervention not captured in this analysis. Adding such a factor would have improved the cost-effectiveness of the program. There are also other health and societal benefits to reducing the unmet need for contraception in LMICs (22, 23) that were not accounted for in this analysis because of its specific scope. Including these would also likely have improved the overall positive result of this program.

Limitations in this evaluation include the dearth of baseline data available for contraceptive use among MWRA in the target population. Health information systems are weak in Pakistan (24), and accurate basic information is often lacking. Although there was a good-faith effort on the part of the implementing partner to obtain accurate information on the level of contraception in the specific target population, it is possible that this was an under- or overestimation. However, the changes to this and other variables would not have a substantive change in the overall result that would change the conclusions of recommending the program for continuation or expansion to similar settings in Pakistan.

Another weakness of the evaluation was the assumption that all services provided for by the voucher program and therefore counted as additional services, with their associated CYPs, attributed to the presence of the SSF. It is possible that a proportion of MWRAs receiving such services would have done so regardless of the SSF program. This assumption may have biased the result toward a more positive outcome: a lower cost per CYP than there may actually be.

## Conclusion

The cost-effectiveness of this implementation of the SSF in 29 districts in Sindh and 3 districts in Punjab between October 2013 and June 2016 was $4.27 per additional CYP compared to business-as-usual from the perspective of the funder. This compares favorably to other interventions with similar objectives and appears affordable for the Pakistan national health-care system. It is therefore recommended to help address the unmet need for contraception among MWRA in these areas of Pakistan by considering a trail implementation in the country more widely.

## Ethics Statement

This research involved the analysis of routinely collected data from patients that were completely anonymous. No additional data were collected. The research was therefore exempted from full Institutional Review Board review in both Pakistan and the United States.

## Author Contributions

EB designed the study, wrote the protocol, performed the analysis, and wrote the first draft of the manuscript. WH and AG collected data on effectiveness of the program and provided information of implementation. SS and IB collected the cost data and helped perform the preliminary analysis on costs. MV designed and supported the overall evaluation and provided guidance on the program’s history and critical revisions to all versions of the manuscript. WH, AG, and SS all had critical input on the design of the study, protocol development, and all versions of the manuscript. All the authors approved the final version of the manuscript.

## Conflict of Interest Statement

The authors declare that the research was conducted in the absence of any commercial or financial relationships that could be construed as a potential conflict of interest.

## Footnotes

^1^This tool measures socioeconomic status in this setting to determine those living in relative poverty (4).

## References

[1] Family Planning 2020. FP2020: Momentum at the Midpoint. Washington, DC: Family Planning 2020 Partnership (2016).

[2] Ngo TD Nuccio O Pereira SK Footman K Reiss K Evaluating a LARC expansion program in 14 Sub-Saharan African countries: a service delivery model for meeting FP2020 goals. Matern Child Health J (2016) 5:26–32. 10.1007/s10995-016-2014-0 PMC5569118 27154524

[3] Khan AA Khan A Javed W Hamza HB Orakzai M Ansari A Family planning in Pakistan: applying what we have learned. J Pak Med Assoc (2013) 63(4 Suppl 3):S3–10. 24386723

[4] National Institute of Population Studies (NIPS). In: NIPS and ICF International, editor. Pakistan Demographic and Health Survey 2012–13. Maryland, USA: Islamabad, Pakistan and Calverton (2013).

[5] Azmat SK Hameed W Hamza HB Mustafa G Ishaque M Abbas G Engaging with community-based public and private mid-level providers for promoting the use of modern contraceptive methods in rural Pakistan: results from two innovative birth spacing interventions. Reprod Health (2016) 13:25. 10.1186/s12978-016-0145-9 26987368 PMC4797360

[6] Jain AK Mahmood A Sathar ZA Masood I. Reducing unmet need and unwanted childbearing: evidence from a panel survey in Pakistan. Stud Fam Plann (2014) 45(2):277–99. 10.1111/j.1728-4465.2014.00389.x 24931080

[7] Gold J Burke E Cissé B Mackay A Eva G Hayes B Increasing access to family planning choices through public-sector social franchising: the experience of Marie Stopes International in Mali. Glob Health Sci Pract (2017) 5(2):286–98. 10.9745/GHSP-D-17-00011 28655803 PMC5487090

[8] Innovation for Poverty Action. Progress Out of Poverty Index. New Haven, CT: Innovations for Poverty Action (2017).

[9] Zakiyah N van Asselt AD Roijmans F Postma MJ. Economic evaluation of family planning interventions in low and middle income countries; a systematic review. PLoS One (2016) 11(12):e0168447. 10.1371/journal.pone.0168447 27992552 PMC5167385

[10] Boddam-Whetham L Gul X Al-Kobati E Gorter AC. Vouchers in Fragile States: reducing barriers to long-acting reversible contraception in Yemen and Pakistan. Glob Health Sci Pract (2016) 4(Suppl 2):S94–108. 10.9745/GHSP-D-15-00308 27540129 PMC4990166

[11] Marie Stopes International. New CYP Conversion Factors. London, UK: Marie Stopes International (2012).

[12] Measure Evaluation. Couple-Years of Protection (CYP) (2017). Available from: https://www.measureevaluation.org/prh/rh_indicators/specific/fp/cyp

[13] Shah NM Wang W Bishai DM. Comparing private sector family planning services to government and NGO services in Ethiopia and Pakistan: how do social franchises compare across quality, equity and cost? Health Policy Plan (2011) 26(Suppl 1):i63–71. 10.1093/heapol/czr027 21729919 PMC3606031

[14] Abbas K Khan AA Khan A. Costs and utilization of public sector family planning services in Pakistan. J Pak Med Assoc (2013) 63(4 Suppl 3):S33–9. 24386728

[15] World Bank. The World Factbook: Pakistan. Washington, DC: World Bank (2016).

[16] Seamans Y Harner-Jay CM. Modelling cost-effectiveness of different vasectomy methods in India, Kenya, and Mexico. Cost Eff Resour Alloc (2007) 5:8. 10.1186/1478-7547-5-8 17629921 PMC1947949

[17] Janowitz J A comparison of cost-effectiveness of contraceptive methods. In: Bratt J Rademacher KH Steiner M, editors. International Conference on Family Planning: Research and Best Practices Kampala, Uganda (2009).

[18] Manzoor K. Cost-effectiveness of the family planning programme in Pakistan. Pak Dev Rev (1994) 33(4 Pt 2):711–26. 12346204

[19] Sathar Z Singh S Rashida G Shah Z Niazi R. Induced abortions and unintended pregnancies in Pakistan. Stud Fam Plann (2014) 45(4):471–91. 10.1111/j.1728-4465.2014.00004.x 25469930 PMC4734376

[20] Naveed Z Shaikh BT Nawaz MA. Induced abortions in Pakistan: expositions, destinations and repercussions. A qualitative descriptive study in Rawalpindi District. J Biosoc Sci (2016) 48(5):631–46. 10.1017/S0021932015000255 26262900

[21] Aslam F Aslam M. Report: a study of morbidity of induced abortion data from women belonging to Karachi, Pakistan. Pak J Pharm Sci (2015) 28(1):255–63. 25553703

[22] Sonnenberg FA Burkman RT Hagerty CG Speroff L Speroff T. Costs and net health effects of contraceptive methods. Contraception (2004) 69(6):447–59. 10.1016/j.contraception.2004.03.008 15157789

[23] Vlassoff M Tsoka M. Benefits of meeting the contraceptive needs of Malawian women. Issues Brief (Alan Guttmacher Inst) (2014) (2):1–8. 26159000

[24] Sajwani A Qureshi K Shaikh T Sayani S. eHealth for remote regions: findings from Central Asia health systems strengthening project. Stud Health Technol Inform (2015) 209:128–34. 25980715

